# Technologiebasierte Interventionen zur Alkoholprävention bei Kindern und Jugendlichen

**DOI:** 10.1007/s00103-021-03338-5

**Published:** 2021-05-05

**Authors:** Silke Diestelkamp, Anna-Lena Schulz, Rainer Thomasius

**Affiliations:** DZSKJ – Deutsches Zentrum für Suchtfragen des Kindes- und Jugendalters, Universitätsklinikum Hamburg-Eppendorf, Martinistr. 52, 20246 Hamburg, Deutschland

**Keywords:** E‑Intervention, Alkohol, Prävention, Kinder, Jugendliche, E‑Intervention, Alcohol, Prevention, Children, Adolescents

## Abstract

**Hintergrund:**

Ein früher Einstieg und der exzessive Alkoholkonsum im Kindes- und Jugendalter erhöhen das Risiko für Krankheit, Abhängigkeit und andere kurz-, mittel- und langfristige Beeinträchtigungen durch z. B. Unfälle, Gewalthandlungen und Konflikte. Face-to-Face-Präventionsansätze zeigen signifikante Effekte auf die Reduktion des Alkoholkonsums. Die Inanspruchnahme durch Kinder und Jugendliche mit riskantem Alkoholkonsum ist jedoch oft gering. Technologiebasierte Alkoholprävention hat das Potenzial, mit kosteneffektiven, standardisierten und niedrigschwelligen Maßnahmen die Zielgruppe zu erreichen.

**Fragestellung und Methode:**

Das vorliegende narrative Review bietet einen Überblick über verschiedene Ansätze technologiebasierter Maßnahmen zur Prävention und Frühintervention riskanten Alkoholkonsums bei Kindern und Jugendlichen sowie deren Wirksamkeit und Einsatzbereiche.

**Ergebnisse:**

Technologiebasierte Alkoholprävention kann in einer Vielfalt von Settings eingesetzt werden, z. B. Schule, Gemeinde, hausärztliche Versorgung oder Klinik. Programme werden häufig via interaktive Website mit oder ohne Einbettung in Face-to-Face-Module, via App oder SMS-Nachrichten umgesetzt. Während die kumulierte Evidenz bei Erwachsenen und jungen Erwachsenen für die Wirksamkeit technologiebasierter Alkoholprävention spricht, ist die Studienlage für Kinder und Jugendliche heterogen.

**Diskussion:**

Der Einsatz von technologiebasierter Alkoholprävention bei Kindern und Jugendlichen bietet theoretisch großes Potenzial im Hinblick auf Zielgruppenerreichung, Kosteneffektivität und Nutzereinbindung. Im Fokus zukünftiger Forschung sollten Studienreplikationen und die Evaluation der Wirksamkeit einzelner Elemente stehen, wie etwa der Individualisierung von Inhalten, der Nutzereinbindung durch multiple Kontaktaufnahmen und des Einsatzes von multimedialen Elementen und Funktionen.

## Abstract (en)

**Background:**

Early onset and excessive alcohol use in childhood and adolescence is associated with an elevated risk of experiencing short-, mid-, and long-term negative consequences caused by, e.g., accidents, violent acts, and conflicts. Face-to-face prevention approaches show significant effects on the reduction of alcohol use. However, service utilization is often low among children and adolescents. Technology-based alcohol prevention has the potential to reach this target group with potentially cost-effective, standardized, and low-threshold measures.

**Aim and method:**

This narrative review provides an overview of different approaches of technology-based interventions for the prevention and early intervention of risky alcohol use among children and adolescents, their effectiveness, and settings for implementation.

**Results:**

Technology-based alcohol prevention can be implemented in a variety of settings, e.g., school, community, primary care, or hospital. Implementation is often realized via websites with or without embedding face-to-face modules, apps, or SMS messages. While the cumulative evidence of the effectiveness of technology-based alcohol prevention is strong for adults and young adults, evidence for the effectiveness among children and adolescents is heterogeneous.

**Discussion:**

Technology-based alcohol prevention has great theoretical potential with regards to reach, cost-effectiveness, and user engagement. Study replications are needed and evaluations of the effects of single elements, such as the individualization of content, user engagement through multiple contacts, and the use of multimedia elements and functions, should be addressed by future research.

## Hintergrund

Alkoholkonsum ist einer der 3 bedeutendsten Risikofaktoren für die weltweite Belastung durch Krankheit [1]. Ein früher Einstieg in den Alkoholkonsum ist mit einem erhöhten Risiko der Entwicklung einer alkoholbezogenen Störung im Lebensverlauf assoziiert [2, 3]. Insbesondere der frühe und exzessive Konsum von Alkohol im Kindes- und Jugendalter geht mit erhöhten Risiken für zum Teil schwerwiegende kurz-, mittel- und langfristige Beeinträchtigungen einher [2, 4–6]. Unfälle, Gewalthandlungen, ungewollte sexuelle Handlungen [4, 7, 8], Konflikte mit Peers, in der Schule, in der Familie und mit der Polizei [6, 9] sowie akute Alkoholintoxikationen [10] sind als kurz- und mittelfristige Folgen riskanten Alkoholkonsums zu nennen, die negative Auswirkungen auf die gesundheitliche, schulische bzw. berufliche und psychosoziale Entwicklung haben können.

Eine effektive zielgruppenspezifische Prävention und Frühintervention riskanten Konsums im Kindes- und Jugendalter sind daher von großer Bedeutung. Die Durchführung von Screenings und Kurzinterventionen (Screening and Brief Intervention, SBI) wird in den S3-Leitlinien zur Behandlung von alkoholbezogenen Störungen im Kindes- und Jugendalter empfohlen [11] und ist auch für die selektive und indizierte Prävention geeignet. Unter dem Begriff „Kurzintervention“ werden Interventionen mit 1–6 Sitzungen von ca. 3–45 min Dauer zusammengefasst [12, 13]. Eine zentrale Barriere für deren Durchführung stellt jedoch die besonders niedrige Inanspruchnahme von Hilfsangeboten durch Kinder und Jugendliche mit riskantem Substanzkonsum dar [14].

Zu den Gründen für die geringe Inanspruchnahme zählen häufig ein geringer Leidensdruck von Jugendlichen mit riskantem Alkoholkonsum, die Furcht vor Stigmatisierung und die subjektive Einschätzung, dass erst eine Suchterkrankung die Inanspruchnahme eines Hilfsangebotes nötig macht. Ein weiterer limitierender Faktor von jugendspezifischen Alkoholpräventionsmaßnahmen ist oft eine mangelnde Erreichbarkeit der Zielgruppe sowie mangelnde Akzeptanz der Maßnahmen durch die Zielgruppe [15]. Aufseiten der Durchführenden stellen eine unzureichende Ausbildung und/oder mangelnde Zeit Barrieren für eine flächendeckende Umsetzung von alkoholbezogenen Präventionsangeboten (z. B. in Schule, Jugendarbeit, Sportvereinen) dar. Auf technologiebasierte Prävention treffen viele dieser Barrieren nicht zu, weshalb ihnen großes Potenzial u. a. in Bezug auf Reichweite, Akzeptanz und Kosteneffektivität zugesprochen wird [16–18]. Das vorliegende narrative Review gibt einen Überblick über Ansätze technologiebasierter Interventionen zur universellen, selektiven und indizierten Prävention riskanten Alkoholkonsums bei Kindern und Jugendlichen sowie über deren Einsatzbereiche und Wirksamkeit.

## Fragestellung und Methode

Nach einer Begriffsklärung und Darstellung der Potenziale und möglicher Limitationen technologiebasierter Alkoholprävention folgt ein Abschnitt, in dem Möglichkeiten der Umsetzung theoriebasierter Präventionsansätze in digitalen Formaten vorgestellt werden. Zur Beurteilung des aktuellen Forschungsstandes zur Wirksamkeit technologiebasierter Alkoholprävention bei Kindern und Jugendlichen wurde eine Literaturrecherche zu Übersichtsarbeiten aus den Jahren 2015 bis 2020 durchgeführt. Die Datenbank PubMed wurde anhand der Suchbegriffe ((((adolescen* OR child* OR youth OR young) AND (intervention OR prevention)) AND (alcohol)) AND (internet OR online)) AND (Review OR Meta analysis) durchsucht. Eingeschlossen wurden systematische Reviews und Metaanalysen, die die Wirksamkeit von alkoholbezogener technologiebasierter Prävention und Frühintervention für Kinder und Jugendliche unter 18 Jahren behandelten. Es folgt ein Abschnitt, in dem Ansätze zur Steigerung der Nutzereinbindung („User-Engagement“) vorgestellt werden. Abschließend werden exemplarisch technologiebasierte Ansätze zur Alkoholprävention bei Kindern und Jugendlichen vorgestellt, die im deutschsprachigen Raum eingesetzt und evaluiert wurden, sowie ein US-amerikanischer Ansatz zur Unterstützung von Hausärzt*innen bei der Durchführung alkoholbezogener Kurzinterventionen bei Kindern und Jugendlichen, der unseres Wissens im deutschsprachigen Raum noch nicht erprobt wurde.

## Ergebnisse

### Technologiebasierte Interventionen zur Prävention

Unter technologiebasierter Prävention werden Angebote verstanden, die z. B. über eine Website, eine App, über SMS oder über sogenannte Wearables, wie z. B. eine Smartwatch, funktionieren. Der Terminus umfasst sowohl E‑ als auch M‑Health-Angebote. Unter dem Begriff E‑Health oder E‑Interventionen werden Interventionen zusammengefasst, die im Gegensatz zu herkömmlichen Face-to-Face-Interventionen internet- oder computerbasiert sind, z. B. Webseiten, Computerspiele, Chats, Foren oder soziale Medien (Facebook, Instagram, Twitter; [19]). Unter M‑Health wird die Nutzung mobiler Endgeräte zur Erkennung und Intervention verstanden, z. B. durch Wearables (Smartwatches, transdermale Pflaster (Patches), Brustbänder, GPS-Tracking; [19]). Wearables und Mobiltelefone können neue Möglichkeiten der Prävention und Frühintervention „in Echtzeit“ eröffnen, indem das „Internet der Dinge“, also die Vernetztheit unterschiedlicher Geräte wie Smartphones, Wearables und anderer internetbasierter Anwendungen, genutzt wird [20].

Technologiebasierte Interventionen zeichnen sich durch eine Reihe von potenziellen Vorteilen aus, wie z. B. die standardisierte Durchführung, die Erreichbarkeit großer und schwer erreichbarer Zielgruppen, eine potenziell hohe Kosteneffektivität, die Umsetzung automatisierter individueller Angebote mit gestuftem Vorgehen (Stepped Care), die Anonymität und damit einhergehend eine geringere Stigmatisierung. Die Möglichkeit zu einer attraktiven Gestaltung durch grafische und spielerische Elemente und die Möglichkeit der zeitlich und inhaltlich selbstbestimmten Nutzung tragen dazu bei, dass technologiebasierte Prävention als besonders niedrigschwellig wahrgenommen wird [16–18].

Zu den potenziellen Limitationen von E‑ und M‑Health-Angeboten kann die technische Umsetzung zählen, die abhängig von dem für die Entwicklung zur Verfügung stehenden Budget ist. Dies betrifft z. B. Präventionsangebote, die Computerspielen (Games) nachempfunden sind. Eine mögliche Simplifizierung therapeutischer Inhalte und eine eingeschränkte nonverbale Kommunikation werden als weitere potenzielle Einschränkungen genannt [16–18]. Die Erreichbarkeit der Zielgruppe, die oben als besonderes Potenzial beschrieben wurde, kann gleichsam eine Herausforderung darstellen, da E‑ oder M‑Health-Angebote, die frei im Internet verfügbar sind, von der Zielgruppe zum Teil schwer gefunden und nicht regelmäßig genutzt werden. E‑ und M‑Health-Angebote können als universelle oder indizierte Prävention in verschiedenen Settings, z. B. in Schule, Beratung, beim Haus- oder Kinderarzt oder in Jugend- und Gesundheitszentren, eingesetzt werden.

### Digitale Umsetzung präventiver Ansätze

In den meisten theoriebasierten digitalen Interventionen wird ein Ansatz sozialer Normen (Social Norms) mit personalisiertem normativen Feedback und Elementen der motivierenden Gesprächsführung (Motivational Interviewing, MI) umgesetzt [18, 21, 22]. Außerdem sind Elemente aus der kognitiv-behavioralen Therapie (CBT), der behavioralen Selbstkontrolle (BSC), den Fähigkeitentrainings (Skills-Training), der sozialkognitiven Theorie und der Theorie geplanten Handelns (Theory of Planned Behavior) in einigen Interventionen vertreten. Die einzelnen Elemente dieser für den Face-to-Face-Gebrauch entwickelten Ansätze eignen sich unterschiedlich gut für die Umsetzung in digitalen Interventionen.

Shingleton und Palfai [21] untersuchten in einem systematischen Review, inwieweit es in E‑Interventionen zur Förderung des Gesundheitsverhaltens gelingt, wesentliche Merkmale und Techniken des MI umzusetzen. Die Autoren schlussfolgern, dass eine motivierende Gesprächsführung auf vielfältige Weise technisch umgesetzt werden kann, z. B. im Rahmen von Chats, Foren, Emojis, Audio- oder Videoelementen oder individualisierten automatischen Konversationen. Besonders häufig wurden die MI-Elemente „Diskrepanzwahrnehmung stärken/Ambivalenz explorieren“, „Selbstverpflichtung stärken“ und „Individualisierung nach Veränderungsmotivation“ in den analysierten Studien umgesetzt. Diese Elemente eignen sich besonders für eine digitale Adaptation, da sie durch Instrumente umgesetzt werden können, die sich digital gut abbilden lassen, z. B. eine Motivationswaage oder die Definition eines Veränderungsplanes. Andere MI-Elemente wurden seltener umgesetzt, wie z. B. offene Fragen stellen, ein MI-kongruenter Umgang mit Widerstand, Partnerschaftlichkeit, Autonomie stärken oder Zusammenfassen.

Die Ergebnisse des Reviews stehen im Einklang mit der Einschätzung, dass digitale Interventionen besondere Stärken in der Umsetzung bestimmter Inhalte aufweisen. Song et al. [23] fanden in ihrem systematischen Review 3 inhaltliche Funktionen von mobilen Interventionen zur Reduktion problematischen Alkoholkonsums, die in der Mehrzahl der Interventionen umgesetzt wurden: Informationsvermittlung, Motivierung und Erinnerungen. Darüber hinaus lassen sich insbesondere Monitoringfunktionen (z. B. Tagebuchfunktionen) und Feedbackfunktionen (z. B. zu individuellen Trinkmustern oder zur Zielerreichung) in E‑ und M‑Health-Interventionen gut umsetzen.

### Wirksamkeit bei problematischem Alkoholkonsum von Kindern und Jugendlichen

Eine große Zahl von Studien stützt die Wirksamkeit von technologiebasierter Prävention und Frühintervention bei problematischem Substanzgebrauch im Erwachsenenalter [18, 23–26]. Auch bei jungen Erwachsenen belegt eine wachsende Anzahl von Studien die Wirksamkeit von technologiebasierten alkoholbezogenen Interventionen in Bezug auf die Reduktion riskanten Konsums und alkoholbezogener Probleme [16, 26–28]. Trotz des oft früh einsetzenden problematischen Alkoholkonsums stammt die Mehrheit der vorliegenden Evidenz für junge Menschen aus Studien mit US-amerikanischen Collegestudierenden. Studien zu Kindern und Jugendlichen sind dagegen rar.

**Figure 1:**
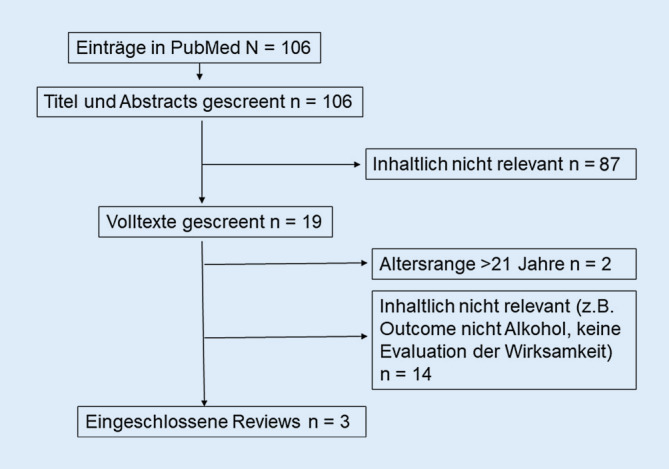

In der PubMed-Literaturrecherche zu aktuellen Übersichtsarbeiten zur Wirksamkeit technologiebasierter Alkoholprävention für Kinder und Jugendliche wurden von den *N* = 106 identifizierten Einträgen *n* = 87 nach dem Screening der Titel und Abstracts ausgeschlossen, weil sie inhaltlich nicht relevant waren. Von den *n* = 19 gesichteten Volltexten wurden *n* = 2 Übersichtsarbeiten ausgeschlossen, weil sie ausschließlich oder überwiegend Studien mit Teilnehmer*innen >21 Jahren beinhalteten. Weitere *n* = 14 Arbeiten erwiesen sich als inhaltlich nicht relevant, weil z. B. die Interventionen nicht auf die Prävention problematischen Alkoholkonsums abzielten oder die Übersichtsarbeiten keine Informationen zur Wirksamkeit der Interventionen behandelten (Abb. 1).

Als relevant wurden *n* = 3 Übersichtsarbeiten identifiziert ([29–31]; Tab. 1). Berichtet werden hier nur die Ergebnisse der Studien in den Übersichtsarbeiten, die sich auf die Prävention problematischen Alkoholkonsums beziehen.

**Table 1:** 

Review	Anzahl der Studien zu Alkoholprävention	Alters-Range	Primärer Endpunkt	Art der Prävention	Setting	Technische Umsetzung	Ergebnisse (Anzahl der Studien mit signifikantem Ergebnis)
Ondersma et al. (2019; [31])	*N* = 20	<18 Jahre	Alkoholkonsum (*n* = 10)	Universell und selektiv	Schule (*n* = 11)	Web- oder computerbasierte Kurzintervention (*n* = 10)	Sign. Reduktion des Alkoholkonsums^a^ (*n* = 12)
			Alkohol- und Substanzkonsum (*n* = 9)		Gesundheitswesen (*n* = 3)	SMS-Nachrichten (*n* = 2)	Sign. Steigerung alkoholbezogenen Wissens (*n* = 1)
			Alkohol- und Tabakkonsum (*n* = 1)		Notaufnahme (*n* = 2)	Soziale Medien (*n* = 0)	Sign. Reduktion alkoholbezogener Konsequenzen (*n* = 1)
					Gemeinde (*n* = 3)	Web- oder computerbasierte Intervention mit mehreren Sitzungen (*n* = 8)	
					Internet (*n* = 1)		
Champion et al. (2016; [30])	*N* = 10^b^	11–18 Jahre	Alkoholkonsum (*n* = 2)	Universell	Schule (*n* = 10)	Webbasierte Kurzintervention (*n* = 2)	Sign. Reduktion des Alkoholkonsums^a^ (*n* = 3)
			Alkohol- und Substanzkonsum (*n* = 5)			Social-Marketing-Intervention (*n* = 1)	Sign. Steigerung alkoholbezogenen Wissens (*n* = 2)
						Web- oder computerbasierte Intervention mit mehreren Sitzungen (*n* = 4)	Sign. reduzierte Intention zum Alkoholkonsum (*n* = 1)
Hopson et al. (2015; [29])	*N* = 17	8–26 Jahre	Alkoholkonsum (*n* = 11)	Universell und selektiv	Schule (*n* = 8)	Web- oder computerbasierte Kurzintervention (*n* = 13)	Sign. Reduktion des Alkoholkonsums^a^ (*n* = 12)
			Alkohol- und Substanzkonsum (*n* = 6)		College (1. Jahr; *n* = 6)	Web- oder computerbasierte Intervention mit mehreren Sitzungen (*n* = 4)	Sign. Steigerung alkoholbezogenen Wissens (*n* = 5)
					Gemeinde (Kind und Elternintervention; *n* = 3)		Sign. Reduktion alkoholbezogener Konsequenzen (*n* = 2)
							Sign. reduzierte Intention zum Alkoholkonsum (*n* = 2)
							Sign. Reduktion einer positiven Einstellung zu Substanzkonsum (*n* = 2)
							Sign. Reduktion der Häufigkeit von Trinkspielen (*n* = 1)

^a^Z. B. Trinkhäufigkeit, Häufigkeit von Binge Drinking (Rauschtrinken), Trinkmenge an einem typischen Konsumtag

^b^In diesem Review wird über *N* = 10 Studien zu *n* = 7 Präventionsprogrammen berichtet

Ondersma et al. [31] stellen in ihrem narrativen Review 21 Studien zur Evaluation digitaler Prävention von Substanzmissbrauch bei <18-Jährigen vor, von denen 20 Studien Alkoholkonsum als Outcome gemessen haben. Die Präventionsprogramme wurden in den Settings Schule (*n* = 11), Gesundheitswesen (*n* = 4), Notaufnahme (*n* = 2), Gemeinde (*n* = 3) und frei im Internet zugänglich (*n* = 1) durchgeführt. Während für motivierende Kurzinterventionen für riskant konsumierende Jugendliche in Settings wie Notaufnahme, Schule, Gemeinde oder Hausarzt vielfach keine signifikanten Effekte auf einen reduzierten Konsum berichtet wurden, zeigten digitale Kurzinterventionen der universellen Prävention, z. B. in Schulen oder Gesundheitszentren, mehrheitlich positive Effekte wie die Reduktion von Alkoholkonsum und/oder alkoholbezogenen Problemen. Interventionen mit mehreren Sitzungen wurden häufig schul- oder gemeindebasiert durchgeführt und bestanden neben digitalen Modulen aus Lehr- oder Beratungseinheiten, in denen die in den digitalen Modulen behandelten Themen vertieft wurden. Während einige Studien positive Effekte auf einen reduzierten Alkoholkonsum und alkoholbezogenes Wissen berichteten, fanden andere Studien diese Effekte nicht. Die Autoren bemängeln die unterschiedliche Aussagekraft der Studien aufgrund von methodischen Limitationen, wie z. B. hohen Drop-out-Raten bei den Nachbefragungen, kleinen Stichproben und unzulänglichem Umgang mit fehlenden Werten in der Datenanalyse.

Champion et al. [30] führten ein systematisches Review zu universellen digitalen Präventionsprogrammen zur Reduktion des Substanzgebrauches bei Kindern und Jugendlichen zwischen 11 und 18 Jahren durch, die in einem randomisiert kontrollierten Studiendesign evaluiert worden sind. Sie analysierten *n* = 10 Studien zu *n* = 7 alkoholbezogenen Präventionsprogrammen, die im Setting Schule durchgeführt wurden. Insgesamt zeigte sich ein heterogenes Bild bzgl. der Wirksamkeit der Programme auf einen reduzierten Alkoholkonsum. 3 Studien berichteten signifikante Reduktionen des Alkoholkonsums in den Interventionsgruppen, darunter 2 Studien zu einer 30-minütigen Feedbackintervention (eCheckUp To Go) und eine Studie zu einem digitalen Alkohol- und Cannabispräventionsprogramm mit 12 Lehreinheiten für Schulklassen (Climate Schools). 2 Studien fanden außerdem eine signifikante Steigerung des alkoholbezogenen Wissens und in einer Studie berichteten die Teilnehmenden der Interventionsgruppe von einer reduzierten Intention, Alkohol zu konsumieren.

Hopson et al. [29] stellen in ihrer Übersichtsarbeit *N* = 17 Studien zu digitalen Alkoholpäventionsprogrammen bzw. kombinierten Substanz- und Alkoholpräventionsprogrammen für Kinder, Jugendliche und junge Erwachsene vor. 8 der beschriebenen Programme wurden in Schulen eingesetzt, 4 davon setzen sich aus mehreren digital vermittelten Unterrichtseinheiten zusammen. 6 Programme richteten sich an Erstsemesterstudierende und wurden an US-amerikanischen Colleges eingesetzt. 3 Programme wurden gemeindebasiert durchgeführt und richteten sich an Eltern-Kind-Dyaden. In 12 der 17 Studien wurden signifikant stärkere Reduktionen des Alkoholkonsums in den Interventionsgruppen berichtet. Weitere Effekte betrafen Steigerungen des alkoholbezogenen Wissens (*n* = 5), die Reduktion alkoholbezogener Konsequenzen (*n* = 2), eine reduzierte Intention, Alkohol konsumieren zu wollen (*n* = 2), die Reduktion einer positiven Einstellung zu Substanzkonsum (*n* = 2) sowie eine Reduktion der Häufigkeit von Trinkspielen (*n* = 1).

Insgesamt verdeutlichen diese 3 aktuellen Übersichtsarbeiten, dass die Datengrundlage zur Beurteilung der Wirksamkeit technologiebasierter Alkoholprävention bei Kindern und Jugendlichen heterogen ist. Umfang, technische Umsetzung, Inhalt und Setting der Durchführung unterscheiden sich zwischen den vorliegenden Programmen teils erheblich, sodass verallgemeinernde Aussagen zur Wirksamkeit nur mit Einschränkungen zulässig sind. Universelle, schulbasierte Programme erscheinen vielversprechend, wobei die Anzahl der Lehreinheiten nicht direkt mit einer gesteigerten Effektivität zusammenzuhängen scheint. Die Ergebnisse für selektive Prävention bereits riskant Alkohol konsumierender Kinder und Jugendlicher sind uneinheitlich und bedürfen weiterer Forschung zur Aufklärung der Bedingungen, die die Wirksamkeit beeinflussen.

### User-Engagement

Die Nutzereinbindung (User-Engagement), d. h. das Finden, Einloggen und die Nutzung einer digitalen Intervention, ist eine zentrale Voraussetzung für deren Wirksamkeit [32]. Vor allem in Bereichen, in denen digitale Interventionen nicht in ein Face-to-Face-Setting eingebettet sind, müssen digitale Interventionen besonders attraktiv und zielgruppenspezifisch in Bezug auf Inhalte, Designs und Nutzungsbedingungen gestaltet sein [32]. 3 der Bereiche, die bereits als in digitalen Interventionen besonders gut umsetzbar beschrieben wurden – Selbstbeobachtung (Self-Monitoring), personalisiertes Feedback und Erinnerungen (Prompts) –, wurden auch als besonders effektiv zur Steigerung der Nutzereinbindung und Wirksamkeit identifiziert [16, 32]. Eine weitere Metaanalyse [33] stützt die Annahme der Effektivität multipler Kontaktaufnahmen über Nachrichten, Erinnerungen oder kurzes Feedback als Ergänzung zu einer Intervention in Bezug auf eine erhöhte Nutzereinbindung.

Erinnerungen können in Form von Mikrointerventionen, wie z. B. einer Kontaktaufnahme via SMS, umgesetzt werden. Sie können (1) vom Nutzer ausgelöst werden, (2) automatisch vorprogrammiert sein (z. B. zu bestimmten Zeiten erfolgen) oder (3) kontextspezifisch ausgelöst werden (z. B. wenn ein GPS-Signal meldet, dass der/die Nutzer*in sich einem zuvor spezifizierten Risikoraum nähert; [34]). Der Einsatz solcher Mikrointerventionen oder Booster bietet die Möglichkeit, Jugendliche in ihrem Lebenskontext mit Interventionsbotschaften zu erreichen (Ecological Momentary Intervention, EMI; [33, 35]). O’Rourke et al. [16] fanden in ihrem systematischen Review, dass insbesondere SMS-basierte Booster bei Jugendlichen und jungen Erwachsenen mit signifikant höherer Nutzereinbindung, einer erhöhten Veränderungsbereitschaft zur Reduktion riskanten Alkoholkonsums sowie mit stärkeren Reduktionen exzessiven Alkoholkonsums assoziiert waren. Eine Metaanalyse von Mason et al. [36] zur Wirksamkeit von substanzbezogenen SMS-basierten Interventionen bei Jugendlichen und jungen Erwachsenen fand, dass SMS-basierte Interventionen mit signifikanten Reduktionen einer Effektgröße von ES = 0,25 im Alkohol- und Tabakkonsum assoziiert waren.

### Beispiele technologiebasierter Alkoholprävention für Kinder und Jugendliche

Ein Beispiel für ein webbasiertes Screening und Frühintervention für riskant Alkohol konsumierende Jugendliche, das u. a. im deutschsprachigen Raum eingesetzt und evaluiert wurde, ist die interaktive, vollautomatisierte motivierende Kurzintervention „WISEteens“ [37]. Ziele der theoriebasierten Intervention sind die Reflexion des Konsumverhaltens, die Auslösung und Stärkung einer Motivation zur Alkoholkonsumreduktion oder -abstinenz und zur Abstinenz von illegalen Drogen. Jugendliche mit einem positiven Screening für riskanten Alkoholkonsum nehmen an der ca. 45-minütigen webbasierten motivierenden Kurzintervention teil. Diese beinhaltet Elemente, wie z. B. normatives Feedback, Reflexion der Vor- und Nachteile des aktuellen und angestrebten Konsums, Förderung der Selbstwirksamkeit und der Veränderungszuversicht sowie Unterstützung beim Aufbau konkreter situationsspezifischer Handlungsskripte. Die Wirksamkeit der im Internet frei verfügbaren Intervention wurde in einer 2‑armigen randomisiert kontrollierten Studie mit 16- bis 18-Jährigen (*N* = 1449) in 4 europäischen Ländern untersucht. Im Vergleich zur Kontrollgruppe berichteten die Teilnehmer*innen der Interventionsgruppe 3 Monate nach Teilnahme an der Intervention von signifikanten Reduktionen des riskanten Alkoholkonsums [37]. Eine Weiterentwicklung der WISEteens-Intervention für Schüler*innen ab 12 Jahren wird zurzeit in einer randomisiert kontrollierten Studie an 5 Standorten in Deutschland im Rahmen der ProHEAD-Studie evaluiert [38]. In Ergänzung zu der webbasierten Kurzintervention erhalten teilnehmende Schüler*innen wöchentliche SMS-Nachrichten über einen Zeitraum von 12 Wochen, in denen zunächst die Trinkintention für das folgende Wochenende erhoben wird. Es folgt ein vollautomatisierter, interaktiver, nach Alter, Geschlecht und Trinkmotiven individualisierter SMS-basierter Dialog mit dem Ziel, die Teilnehmenden zu einem risikoarmen Alkoholkonsum zu motivieren.

Die Arbeitsgruppe um Haug und Kolleg*innen [39] entwickelte eine internet- und SMS-basierte Intervention zur Reduktion riskanten Alkoholkonsums für Schüler*innen. Nach einer Eingangsbefragung zum Trinkverhalten mit Feedback erhielten die Teilnehmer*innen individualisierte SMS-Nachrichten über einen Zeitraum von 3 Monaten. Die SMS-Nachrichten bezogen sich auf die Motivation zu risikoarmem Konsum, alkoholbezogene Ergebniserwartungen und soziale Norm sowie auf alkoholbezogene Selbstwirksamkeit und Planungsprozesse. In einer clusterrandomisiert kontrollierten Studie berichteten Studienteilnehmer*innen signifikante Reduktionen der Prävalenz des Rauschtrinkens mit besonders starken Reduktionen in der Gruppe der exzessiv Konsumierenden. Unter den Studienteilnehmer*innen mit kürzlichem Rauschtrinken wurde zusätzlich die Wirksamkeit einer SMS-basierten individualisierten Mikroplanungsintervention (wenn Situation *x* eintritt, werde ich Strategie *y* anwenden) untersucht, die zu Hochrisikozeiten für riskanten Konsum versendet wurde und zum Ziel hatte, riskanten Alkoholkonsum mit Freunden oder beim Ausgehen zu reduzieren. Die Studienergebnisse zeigten, dass Jugendliche nach der Teilnahme an der Planungsintervention durchschnittlich 1 Standardgetränk weniger konsumierten [40].

Ein Beispiel für einen tabletgestützten Präventionsansatz mit Screening und Kurzintervention im Hausarztsetting wurde am Boston Children’s Hospital entwickelt und evaluiert [41]. 12- bis 18-jährige Kinder und Jugendliche füllten im Rahmen einer Vorsorgeuntersuchung im Wartebereich am Tablet einen Screeningtest auf riskanten Alkoholkonsum aus. Sie erhielten daraufhin ein personalisiertes Feedback zu ihren Konsumangaben sowie Zugang zu interaktiven, psychoedukativen Seiten, die über gesundheitliche Risiken aufklären, die mit riskantem Alkoholkonsum assoziiert sind. Basierend auf dem Testergebnis führte der/die behandelnde Ärzt*in eine leitfadengestützte 2‑ bis 3‑minütige Kurzintervention durch. In einer randomisiert kontrollierten Studie wurden Machbarkeit und Wirksamkeit der tabletgestützten Kurzintervention (e-SBI) im Vergleich zu einer herkömmlichen Kurzintervention (SBI) ohne Tabletunterstützung untersucht. Die Studienergebnisse zeigten, dass Ärzt*innen in der tabletunterstützten Gruppe signifikant häufiger Kurzinterventionen durchführten, als Ärzt*innen, die ohne Tablet arbeiteten. Jugendliche, die vor der Vorsorgeuntersuchung bereits Alkohol konsumiert hatten, gaben nach der tabletgestützten Kurzintervention einen längeren Zeitraum bis zum nächsten Konsum an.

## Fazit

Technologiebasierte Alkoholprävention für Kinder und Jugendliche kann auf vielfältige Weise umgesetzt werden und hat das Potenzial, als anonymes, oft kostenloses, zeitlich flexibel nutzbares und damit sehr niedrigschwelliges Angebot große und möglicherweise schwer erreichbare Nutzergruppen zu erreichen. Darüber hinaus bieten digitale Präventionsprogramme den Vorteil einer standardisierten Durchführung und Möglichkeiten der Individualisierung von Inhalten, die in analogen Programmen schwer umsetzbar sind. Technologiebasierte Prävention kann insbesondere für die Zielgruppe der Kinder und Jugendlichen attraktiv sein, da die Nutzung webbasierter Inhalte und die Informationssuche nach gesundheitsbezogenen Themen im Internet bei ihnen weitverbreitet sind [42].

Die Einsatzbereiche für technologiebasierte Prävention sind vielfältig. Universelle Präventionsangebote sind oft in den Kontext Schule eingebettet, in die Gesundheitsversorgung oder in gemeindebasierte Programme. Selektive Präventionsangebote adressieren Jugendliche, die bereits riskant Alkohol konsumieren, und können z. B. im Notfallsetting oder in der Jugendarbeit angesiedelt sein. M‑Health-Interventionen bieten großes Potenzial, Jugendliche mit multiplen Mikrointerventionen in ihrem Lebenskontext zu erreichen und dadurch die Nutzereinbindung und Effektivität zu erhöhen.

Aktuell gibt es wenige evidenz- und theoriebasierte Angebote für die Altersgruppe der Kinder und Jugendlichen, die das theoretisch aufgezeigte Potenzial digitaler Prävention ausschöpfen. Ein Grund hierfür ist, dass digitale Präventionsangebote für diese Zielgruppe stets auch mit anderen Inhalten digitaler Medien, wie z. B. Games oder sozialen Medien, konkurrieren, die die Gewohnheiten von Nutzer*innen in Bezug auf grafische Darstellung und Unterhaltungswert der Inhalte formen. Entwickler von evidenz- und theoriebasierten digitalen Präventionsangeboten, die im wissenschaftlichen Bereich meist ein kleineres Budget zur Verfügung haben als z. B. die Games-Industrie, stellt dies vor die Herausforderung, mit kreativen Mitteln attraktive Angebote für Kinder und Jugendliche zu entwickeln. Interessante Ansätze und Prinzipien können hier z. B. den sogenannten Serious Games for Health entlehnt werden. Wichtig ist in diesem Zusammenhang eine für die Nutzer*innen eindeutige Kennzeichnung evidenzbasierter Angebote.

Während die kumulierte Evidenz bei Erwachsenen und jungen Erwachsenen für die Wirksamkeit technologiebasierter Alkoholprävention spricht, ist die Studienlage für Kinder und Jugendliche insgesamt noch lückenhaft und heterogen. Dies ist vor allem auf die geringe Anzahl von Studien zurückzuführen, die bislang mit Minderjährigen durchgeführt wurden. Da die Umsetzung technologiebasierter Interventionen in den vorliegenden Studien häufig sehr unterschiedlich ist (z. B. 30-minütige webbasierte Intervention vs. wöchentliche SMS über 3 Monate) und darüber hinaus z. B. der Grad der Interaktivität und Individualisierung, die eingesetzten theoriebasierten Elemente und das Setting variieren, ist die Datengrundlage zur Bewertung der Wirksamkeit technologiebasierter Alkoholprävention für diese Zielgruppe aktuell heterogen.

Bei dem Einsatz digitaler Interventionen sollte stets der mögliche Nutzen gegenüber den potenziellen Kosten oder Risiken abgewogen werden. Dem Nutzen von beispielsweise einer Echtzeitübermittlung der Blutalkoholkonzentration an eine App sind die Einschränkungen der Privatsphäre, der individuellen Selbstbestimmung und des Datenschutzes gegenüberzustellen. Die Frage der Datensicherheit muss bei der Erhebung und Übermittlung sensibler persönlicher Daten immer transparent und umfassend geklärt sein und ein Missbrauch (z. B. durch eine Weitergabe an Krankenkassen) ausgeschlossen sein. Das Risiko einer Entwicklung oder Verstärkung einer Online- oder Computerspielsucht durch die Nutzung digitaler Alkoholpräventionsangebote wird hingegen als gering eingeschätzt, da Elemente, die eine Suchtentwicklung begünstigen können, in technologiebasierten Alkoholpräventionsprogrammen typischerweise nicht vorkommen [43].

Zukünftige Forschung sollte u. a. durch Studienreplikationen und die Evaluation einzelner Elemente die systematische Beforschung der Bedingungen für wirksame technologiebasierte Alkoholprävention für Kinder und Jugendliche vorantreiben. Insbesondere das Potenzial zur Individualisierung von Inhalten, zur Steigerung der Nutzereinbindung durch multiple Kontaktaufnahmen und den Einsatz multimedialer Elemente sowie die stärkere Verankerung der Programme in den Lebenskontexten der Teilnehmenden durch Ecological Momentary Interventions sollten im Fokus zukünftiger Studien stehen.
