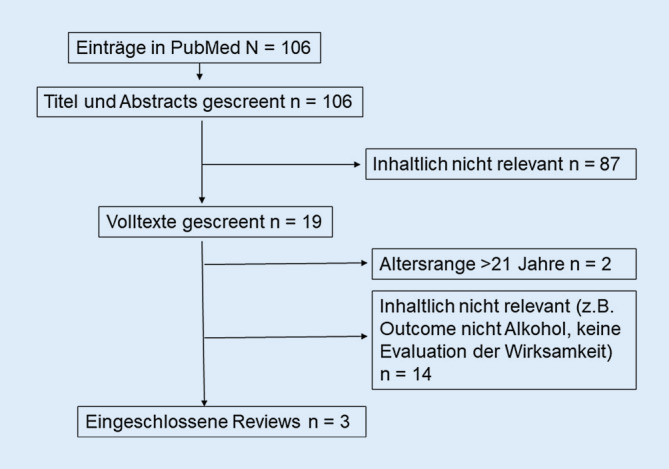# Erratum zu: Technologiebasierte Interventionen zur Alkoholprävention bei Kindern und Jugendlichen

**DOI:** 10.1007/s00103-021-03365-2

**Published:** 2021-06-11

**Authors:** Silke Diestelkamp, Anna-Lena Schulz, Rainer Thomasius

**Affiliations:** DZSKJ – Deutsches Zentrum für Suchtfragen des Kindes- und Jugendalters, Universitätsklinikum Hamburg-Eppendorf, Martinistr. 52, 20246 Hamburg, Deutschland

**Erratum zu:**

**Bundesgesundheitsbl 2021**

10.1007/s00103-021-03338-5

In der ursprünglichen Originalfassung des Artikels wurde leider Abb. 1 nicht korrekt wiedergegeben. Die korrigierte Abbildung sieht wie folgt aus. Die Abbildung wurde im Originalbeitrag korrigiert.

**Figure 1:**